# Successful Case of Extirpation of the Uterus

**Published:** 1855-06

**Authors:** 


### *Successful Case of Extirpation of the Uterus.*

By G. Kimball, M. D., Lowell, Mass.

The following case is one remarkable in every respect. While it shows that even so formidable an operation as extirpation of the uterus may result successfully, it by no means proves that it can be often justifiable. The history of cutting operations upon the uterus is marked by disaster. In this case it was, perhaps, the extreme anæmia of the patient which insured recovery.—Ed. Buff. Med. Jour.

In furnishing a report of this successful case of removal of a diseased uterus, I have to acknowledge myself embarrassed somewhat from the want of a more perfect statement of details than I have been able to procure. Such a statement had been promised me by the physician in attendance after the operation. It has, however, never yet come to hand, and in despair of ever receiving it, I am now under the necessity of furnishing a report much less complete than I could have desired, relying mainly, for the essential materials of it, upon statements and details gathered from the several notes received before the operation, and during the patient's recovery.

The following quotation I make from a letter received from the attending physician, Dr. A. Skinner—dated

*Vernon, Ct., Aug.* 16, 1853.

"Dr. Kimball: Dear Sir,—Mrs. T., of this town, some time since called my attention to a small tumor situated in the abdomen, on the left side, and as low down as the region occupied by the uterus. This struck me at first as being possibly of serious importance, and requiring special attention. Some few month passed on, and I consulted Prof. Knight, of New Haven, regarding the case. But he added nothing by way of explaining the real nature of the disease, nor did he propose any new treatment of it. Some months after, Dr. Knight was again consulted—still no improvement. Up to this time everything in the form of prescribed remedies has failed in retarding the growth of the tumor, till now it fills a large place in the abdomen.

"No great inconvenience attends the *size* of the tumor, but the trouble is from hemorrhage during the period of menstruation. Every month a large quantity of blood is lost, reducing the patient extremely—even hazarding her life. Now the question is, can this be a suitable case for the operation of ovariotomy? Is not the uterus implicated in the disease? The tumor is movable, and, I should think, no very firm attachments had formed. But whence this profuse hemorrhage, if not from the uterus? The patient is 34 years old, and at the commencement of the disease was in robust health."

In reply to this statement, I could only remark that the account given of the case was characteristic of uterine, rather than ovarian disease; yet with this view even, I was not prepared to pronounce it altogether beyond the reach of remedy. On the contrary, rather than give up the case as utterly hopeless, I would propose, as a last resort, the removal of the uterus itself.

In accordance with this suggestion, I was requested to visit the patient at her residence. This I did on the 1st of September, 1853. The suspicions previously entertained regarding the nature of the disease in question, were now fully confirmed, as the facts of the case came to be better known by personal examination. The first aspect of the patient indicated, most unequivocally, an extreme case of *anæmia*. She lay in bed, upon her back, unable to sit up or turn upon her side without help. She had but just rallied from her last attack of hemorrhage, which had been frightfully severe. Another similar attack, if allowed to occur, was looked forward to as an event certain to be fatal. And in due course, this event was now liable to happen at any moment.

Upon examining the tumor, it was found, as had been previously stated, to occupy a very considerable space in the centre of the abdomen. Its form was globular—surface perfectly regular—movable from one side to the other—evidently unattached by adhesions—elastic, without the least sign of containing fluid, yet less solid in its feel than if it had been a more fleshy substance. Its diameter apparently about seven inches.

Examined per vaginam, the neck of the uterus was found in its natural condition, both in position and size. The os uteri open rather more than natural; a sound readily passed up some four or five inches. The enlarged and diseased portion of the organ could not be reached by the forefinger—the entire bulk of the tumor lay in the abdominal cavity.

Without knowing the actual state of the case, one would have judged, from the appearance of the abdomen, that it was a case of pregnancy six months advanced. No lesion, organic or functional, of any other organ, could be detected. Indeed, but for this one difficulty, there seemed no hindrance to the recovery and enjoyment of perfect health.

The important question was now raised, whether the case was one that promised any chance of relief from a surgical operation? The operation proposed was the removal of the uterus by section through the abdominal walls. Extraordinary and hazardous as this suggestion seemed, the feeling was unanimously and unhesitatingly expressed, by every one present at the consultation, that this procedure offered the only possible chance of saving the patient from impending death. This conclusion was no sooner made known to the patient, than it was readily assented to—both she and her husband claiming that a chance of life by an operation, however small that chance might be, was better than the certainty of a speedy death.

The patient was now put in readiness for the operation by being placed on a properly elevated table, and brought under the influence of chloroform. Upon exposing the abdomen, and observing the small size of the patient, it appeared quite evident that in order to dislodge the tumor *entire*, it would be necessary to extend an incision from the ensiform cartilage to the pubis. But rather than do this, it was thought better to expose a part only of the tumor, and see what could be done by way of *enucleating* the diseased portion of it—thus reducing its bulk so as to allow its being drawn out through a comparatively small opening. Accordingly, an incision was made through the *linea alba* directly over the most prominent portion of the tumor, exposing it to the extent of about four inches. Another cut of less extent, through the uterine walls, brought to view the fibrous mass within. Observing that no bleeding followed this procedure, this last incision was prolonged to an extent corresponding with that through the parietes. Through this opening a portion of the diseased mass, thus exposed, was suddenly and forcibly extruded, seeming, at first, as if a little additional force would be sufficient to dislodge it entirely from its connections. Attachments, however, firmer and more extensive than had been anticipated, rendered this part of the operation rather difficult; but being finally accomplished, and the uterus becoming at once greatly diminished in bulk, it was readily drawn out from the abdominal cavity, conformably with the plan adopted in the outset, and placed in the hands of an assistant.

A straight, double-armed needle was now passed through the organ in an antero-posterior direction, as low down as the supposed point of its junction with the neck, this part being, of course, left intact as regards its relation with the vagina. By this plan of appropriating to each lateral half a separate ligature, there was no great difficulty in making sure against all chance of subsequent hemorrhage; a consideration of great importance, in view of what might otherwise be very liable to happen.

The remaining part of the operation was very simple, and easily accomplished. It consisted of a mere amputation of the diseased structure by a single straight incision, carried across from one side to the other, and as near to the ligatures as was consistent with their secure attachment.

The parts having now been made as clean as possible, the wound through the parietes was brought together, and its edges secured with four sutures. Adhesive strips, and a compress wet with warm water and laudanum, completed the dressing.

The operation was somewhat protracted, lasting nearly or quite forty minutes; yet it was not accompanied or followed by any extraordinary or alarming degree of exhaustion. The amount of blood lost did not exceed four ounces.

After being laid in bed, the patient was troubled with nausea, and occasional vomiting, which continued for two or three hours. This, however, was probably the effect of chloroform merely. Upon its ceasing, an urgent desire, without the ability, to evacuate the bladder, came on, together with a severe pain in the lower part of the back. The first difficulty was readily relieved by the use of the catheter, the latter by a half-grain dose of morphine—which seemed not only to quiet the pain, but to induce what was then considered a comfortable night's rest.

For the subsequent history of the case, I am obliged to quote from letters received from time to time from the attending physician, Dr. Skinner.

On Saturday, two days after the operation, he writes as follows: "At 12 o'clock yesterday I was called to see our patient, and found her vomiting severely. Directed an enema of starch and laudanum, with counter-irritation over the stomach. This succeeded in checking the vomiting very soon. Spent the following night with her, and for the most part of the time she was quiet, and when disturbed at all, it was from nausea. Some fullness of the abdomen, with a little tenderness."

"Tuesday, September 6 (sixth day). We find our patient this morning (8 A. M.) comparatively comfortable. Monday, there was much tympanitis and tenderness of the abdomen. There had been considerable nausea the evening previous, and occasional vomiting. Two mild laxative enemas were given, but no evacuation of the bowels followed. Average pulse 116, and somewhat irregular.

"Last evening another laxative enema was given; and a few hours after, still another. This last was soon followed by a good looking movement. Since this, there has been less restlessness. Starch and laudanum injections have been duly kept up. Less flatulence, and with the exception of two paroxysms of vomiting (one since I commenced writing this morning) the symptoms are generally more favorable. Let me add, that during last night there was some fever, face flushed, pulse 125. This morning some pus appeared at the lower part of the incision."

"Thursday morning, Sept. 5th.—Our patient is still alive and rather com fortable. Nausea and vomiting have been the worst symptoms since opera tion. Bowels have not moved since last Thursday. Tympanitis gradually improving. Pulse 100 to 120. Not much febrile action. We allow her a little very weak broth. We have succeeded in getting the full effect of opium by using laudanum injections—the only way opium could be tolerated."

From the date of the above, till January following, accounts of regular improvement were received as often as once every two or three weeks. On the 12th January, Dr. Skinner wro e as follows:

"O r patient remains much the same as when I last wrote. She is able to walk about the house, and looks nearly well. Countenance good; pu'se strong; appetite good enough; bowels free; in short, everything about her *right*, except what is produced by the irritation from her *ligatures*."

March 1st, six months after operation, another communication was received, in which the ligatures are again alluded to as still attached, and causing considerable annoyance from mere local irritation. Again directions were given to apply still more force. This was promptly done; yet the ligature remained firm.

Early in May following, I visited my patient at her residence, and found, as her physician had previously stated, "everything all right, except the irritation produced by the ligatures." Her personal appearance had so changed that I could hardly believe her to be the identical person I had operated on eight months previous. The recovery of flesh and strength—the healthy, florid color of the cheeks—good appetite and perfect digestion, all indicated the return of robust health.

The ligatures, however, still remained an annoyance, producing a good deal of discomfort, particularly in the exercise of riding and walking. Another attempt to remove them was again unsuccessful, and from the pain that always followed these efforts, it was thought advisable rather to allow them to remain attached for an indefinite time longer, than to subject the patient to repeated failures. This conclusion seemed reasonable and safe, from the fact that their presence was looked upon as a mere *inconvenience*, and not implying any danger.

This visit, as stated above, was made early in May, eight months subsequent to the operation. From that time to the present, my further knowledge of the case has been only of an indirect character, yet quite satisfactory. From several individuals coming from the immediate neighborhood of the patient (one of them recently), I learn that the operation is spoken of as perfectly successful, and the patient herself restored to health.

The above case is the only one, I believe, as far as can be ascertained from the records of surgery, where the operation for the removal of the uterus, by what is termed the hypogastric method, has been successful.

M. Langenbeck, of Gottingen, uncle of his distinguished namesake, Prof. Langenbeck, of Berlin, according to the report of a case published by his son in 1813, extirpated the uterus *per vaginam*, and the patient recovered. This, however, was a case of *inverted uterus*. In the two or three other instances where the operation has been effected by section through the abdominal walls, the cases have resulted fatally. In one of these, by Mr. Meaths, of Manchester, Eng., the operation was begun with the view of removing a diseased ovary. The exposure of the tumor, however, disclosed at once an error in diagnosis, showing that the disease in question was not ovarian, but uterine. The surgeon deemed it expedient, however, under the circumstances, to proceed in the operation, and effected the complete ablation of the organ diseased.

I have myself performed this operation in three instances. In one instance, as has been already shown, the result was successful. But in bringing before the profession and the public an account of an operation, the result of which I claim as singular, so far as the record shows, I should consider myself unjust, and culpably indifferent to my professional obligations, were I to withhold the fact that in two other instances of uterine extirpation, I have had the misfortune to lose my patients.

In my first operation, the circumstances attending it were very similar to those named in Mr. Meaths's case—that is, the operation was begun with the view of removing a diseased ovary, and terminated in the extirpation of the uterus. Though feeling well assured in this case as to the correctness of my opinion regarding the nature of the disease I was about to encounter (an opinion, too, which, so far as I know, was concurred in by each of the several medical gentlemen present,) my first incision through the abdominal parietes revealed at once the unexpected yet unmistakable fact that the tumor in question was no other than an enormous, irregular, lobulated structure; the uterus itself being the only organ involved. My determination, in this aspect of things, was to desist from further prosecuting the operation; but upon consultation, another judgment prevailed, and it was finally concluded by a complete extirpation of the diseased mass, and with it, also, the whole of the organ with which it was connected. This patient survived the operation ten days. For the first six days the symptoms were comparatively mild —so much so, as to afford considerable hope of recovery. On the seventh day, however, the aspect of things changed for the worse; and on the tenth day, as before stated, the case terminated in the death of the patient.

The second fatal case was the third, as well as last, in the order of time. The motives inducing me to operate in this instance were substantially the same as those stated in connection with the *second* case that had just resulted favorably, with this additional and important fact, that it was now shown conclusively and satisfactorily that the extirpation of the uterus was by no means a *necessarily* fatal operation. The case, however, terminated unfortunately. The patient died on the third day; and upon post-mortem examination, it was shown, but too clearly, that a ligature had slipped, and a hemorrhage in consequence was the immediate cause of death. But for this accident, there were as good reasons for expecting a good result as in the case immediately preceding it.

The foregoing cases make up the amount of my experience as regards this formidable operation; and it will be observed that these cases all relate to one form of disease, viz, fibrous growths within the walls of the uterus.

Many other instances of a similar character have fallen under my observation during the last fifteen months; but in none of them were there present those conditions which properly suggested a resort to an operation. The cases where such a procedure would be proper, are unquestionably rare; yet my conscientious belief is, that cases now and then do occur where the extirpation of the womb is clearly justifiable and expedient. Moreover, the operation, desperate as it is, seems to be not merely one which the patient is fairly entitled to, but one which the surgeon, upon request, may feel himself *bound*, as a matter of duty, to perform, so long as by so doing he may *possibly* save the life of his patient, while otherwise he is sure to see her pass speedily to the grave. April, 1855.—*Boston Med. and Surg. Journal.*

